# Comparison of endovascular treatment outcomes in stroke patients with cardioembolic or intracranial atherosclerosis-predisposed large vessel occlusion

**DOI:** 10.3389/fneur.2025.1660804

**Published:** 2026-01-19

**Authors:** Fergane Mehmedova, Zehra Uysal Kocabaş, Özlem Aykaç, Hasan Bayındır, Atilla Ozcan Ozdemir

**Affiliations:** 1 Basaksehir Cam and Sakura City Hospital, Istanbul, Türkiye; 2 Department of Neurology, Eskisehir Osmangazi University, Eskisehir, Türkiye; 3 Neurology Clinic, Ankara City Hospital, Ankara, Türkiye

**Keywords:** cardiogenic embolism, clinical outcome, endovascular treatment, intracranial atherosclerotic disease, mTICI

## Abstract

**Objective:**

The benefit of endovascular treatment (EVT) in patients with acute ischemic stroke due to large vessel occlusion is now well established. EVT is highly effective for treating embolic occlusions; however, there remains ongoing debate regarding the optimal management of underlying intracranial atherosclerotic disease (ICAD). Current approaches often involve a combination of best medical therapy and mechanical rescue strategies, such as intracranial angioplasty or stenting. In this study, we aimed to compare EVT outcomes between patients with ICAD-related strokes and those with cardioembolic strokes.

**Materials and methods:**

The study was designed retrospectively. Data of patients admitted to the stroke center were analyzed. The results of the ICAD group were compared with the cardioembolic stroke group. Demographic characteristics, comorbidities, medications, IV rtPA use before EVT, contraindications, radiologic imaging results, and mechanical thrombectomy results were evaluated. Modified Rankin Score (mRS) results at discharge and 3 months were analyzed. At the end of 3 months, those with mRS 0–2 were included in the good outcome group, and those with mRS 3–6 were included in the poor outcome group. In this study, propensity score matching (PSM) was implemented. The logistic regression model was used.

**Results:**

A total of 349 patients were included, with 12% classified in the ICAD group and 88% in the cardioembolic group. Through PSM, 40 matched patients were successfully identified in the cardioembolism group, corresponding to 42 patients in the ICAD group. During EVT, dissection (OR: 1.105, 95% CI: 1.002–1.219) and reocclusion after EVT (*p* = 0.002) rates were statistically significant in the ICAD group. No significant difference in the rate of symptomatic intracerebral hemorrhage (sICH) was observed between the groups (*p* = 0.892). The ICAD group showed higher rates of failed recanalization (mTICI 0–2b) and worse 3-month mRS scores (mRS 3–6) compared to the cardioembolic group.

**Conclusion:**

In this comparative study of EVT outcomes in ICAD-related and cardioembolic strokes, no significant difference was seen in the rate of post-procedural hemorrhagic complications. It was observed that 3-month poor outcome rates were higher in acute stroke patients with ICAD compared to cardioembolic strokes. We revealed that patients with ICAD presenting with acute ischemic stroke demonstrated higher rates of complications (dissection) and lower recanalization rates following EVT. These results highlight the need for tailored therapeutic strategies and careful procedural planning in patients with ICAD to improve clinical outcomes.

## Introduction

Endovascular therapy (EVT) based on mechanical thrombectomy has become the gold standard for stroke patients with acute large vessel occlusion. Intracranial atherosclerotic disease (ICAD) causing thrombosis *in situ* is gaining importance, as well as thromboembolic occlusions among etiologic causes in stroke patients with large vessel occlusion. Patients with ICAD-related large vessel occlusion have a high thrombogenic status associated with acutely inflamed atheromatous plaque. Numerous studies have shown good treatment responses in patients with acute stroke of cardioembolic etiology undergoing EVT. In patients with ICAD-related acute large artery occlusion, EVT has been reported to significantly affect success rates and cause worse outcomes (1–5).

In this study, we aimed to compare the outcomes of patients with acute large vessel occlusion due to cardioembolic etiology who underwent endovascular treatment (EVT), with those of patients who experienced stroke in the context of ICAD.

## Materials and methods

### Study design

The study was designed as a single-center, cross-sectional and retrospective study. The data of acute ischemic stroke patients admitted to Eskişehir Osmangazi University Faculty of Medicine Stroke Center between 01.01.2016–01.08.2022 were evaluated by retrospectively reviewing the interventional procedures data of the center. The data of patients with cardioembolic stroke and intracranial atherosclerotic disease (ICAD) according to TOAST (The Trial of Org 10,172 in Acute Stroke Treatment) were collected. The results of the two groups were compared.

### Data collection

Demographic characteristics, comorbidities, medications, National Institutes of Health Stroke Scale Score (NIHSS), intravenous recombinant tissue plasminogen activator (IV rtPA) use, contraindications and temporal parameters were evaluated. Radiological imaging results [multimode non-contrast computed tomography (CT) and CT angiography] were retrospectively reviewed, and the Alberta Stroke Program Early Computed Tomography Score (ASPECT), hyperdense artery finding, localization of large vessel occlusion, collateral scores TAN and modified TAN were recorded. In mechanical thrombectomy, procedure times, techniques used (aspiration, isolated stent, or combined techniques), the total number of intracranial procedures, and recanalization results were evaluated with modified thrombolysis in cerebral infarction score (mTICI), intra-arterial rtPA administration, balloon angioplasty, use of permanent stents, angio reports, and imaging records were retrospectively reviewed and images were reviewed and recorded individually. After mechanical thrombectomy, procedures with mTICI 0–2b recanalization were recorded as poor results, while procedures with mTICI 2c and 3 recanalization were recorded as good results.

### Procedure complications

Hemorrhage in the infarct area on control imaging after EVT can range from minor petechial hemorrhages to major hemorrhages. The presence of intracranial hemorrhage in the first 24 h after EVT and types of hemorrhage (type 1 and type 2 petechial hemorrhage, type 1 and type 2 hematoma) were recorded by retrospective review of computed tomography images. Patients with clinical progression (≥ 4 increase in NIHSS score) were grouped as symptomatic and patients without clinical progression were grouped as asymptomatic. In addition, other presedural complications and conditions that developed during EVT (vasospasm, dissection, distal embolism and presence of reocclusion) were noted by retrospectively reviewing angiography images and reports.

### Outcome assessment

The modified Rankin Score (mRS) recorded in the outpatient clinic notes was used to evaluate the 3-month clinical status (disability status) of the patients. Patients with mRS 0–2 were included in the good clinical outcome group, whereas patients with mRS 3–6 were included in the poor clinical outcome group.

### Statistical analysis

Data were analyzed by IBM SPSS v29. The conformity of the data to normal distribution was examined by Kolmogorov Smirnov and Shapiro Wilk tests. Independent Two Sample t test was used to compare normally distributed data in paired groups and Mann Whitney U test was used to compare non-normally distributed data. Yates correction, Fisher’s Exact test, Pearson Chi- Square test were used to compare categorical data and Bonferroni Corrected Z test was used for multiple comparisons. Analysis results were presented as frequency (percentage) for categorical variables, mean ± standard deviation, and median for quantitative variables.

To mitigate potential biases due to baseline counfounders, propensity score matching (PSM) was implemented. Propensity scores were calculated using a logistic regression model based on the following covariates: age, gender, hypertension, diabetes mellitus, obesity, active smoking status, prior use of anticoagulants, ASPECT score, and NIHSS score at the time of emergency admission.
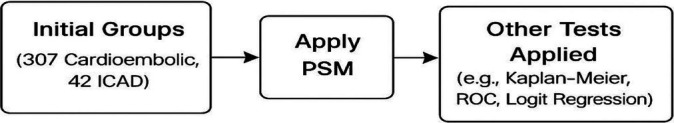


PSM was applied to improve the comparability between the ICAD group (*n* = 42) and the cardioembolism group (*n* = 307). This matching process enabled a robust and balanced comparative analysis between the two groups, minimizing potential confounding biases. After applying PSM to a randomly selected sample of 82 patients, baseline confounders between the ICAD and cardioembolic groups were controlled, allowing for valid comparisons through matched cohorts with similar baseline characteristics.

Ordinal regression was used to explain the correlation between the mRS and mTICI scales. Kaplan–Meier survival, ROC analysis and logistic regression were used to compare ICAD vs. Cardioembolism groups after PSM. The significance level was taken as *p* < 0.050.

## Results

### Patients’ characteristics

A total of 349 patients were included in the study before PSM. The mean age of the patients was 65.01 ± 12.84 years. 188 (53.9%) of the patients were female. According to the TOAST classification, 42 (12%) patients were in the ICAD group, and 307 (88%) patients were in the cardioembolic stroke group. The results of ICAD and cardioembolic stroke groups were compared (Tables 1, 2). Hyperdense artery finding was statistically significant between the groups (*p* = 0.008). While the rate of hyperdense artery findings was 46.3% in the ICAD group, this rate was 68.8% in the cardioembolic stroke group. A statistically significant correlation was obtained between anterior and posterior system occlusions according to the groups (*p* < 0.001). While the rate of anterior system occlusions was 57.1% in the ICAD group, this rate was 94.8% in the cardioembolic stroke group. The rate of posterior system occlusions was 42.9% in the ICAD group and 5.2% in the cardioembolic stroke group.

**Table 1:** Comparison of quantitative parameters according to intracranial atherosclerotic disease and cardioembolic stroke groups.

Parameters	ICAD Group		Cardioembolic Group		All patients		*p* value
	Mean ± S.d	Median (Min–Max)	Mean ± S.d	Median (Min–Max)	Mean ± S.d	Median (Min–Max)	
Age (years)	63.64 ± 12.2	66 (35–84)	65.2 ± 12.94	66 (24–87)	65.01 ± 12.84	66 (24–87)	0.336**
Baseline NIHSS score	18.74 ± 6.3	18 (7–35)	16.34 ± 5.17	16 (2–32)	16.63 ± 5.36	17 (2–35)	0.006*
Symptom-to-Injection Time (min)	130.29 ± 64.14	126 (0–250)	131.98 ± 59.22	125 (0–262)	131.76 ± 59.62	125 (0–262)	0.914*
Door -to-imaging time (min)	37.42 ± 115.75	17 (2–660)	17.54 ± 15.78	14 (0–119)	21.3 ± 52.24	14.5 (0–660)	0.149**
Door-to-Injection Time (min)	49 ± 17.58	49 (0–76)	56.03 ± 29.75	50.5 (0–186)	55.13 ± 28.53	50 (0–186)	0.642**
ASPECT score	8.63 ± 1.44	9 (4–10)	8.76 ± 1.33	9 (4–10)	8.75 ± 1.34	9 (4–10)	0.917**
Door-to-femoral puncture time (min)	129.36 ± 124.08	100 (36–780)	97.16 ± 47.62	90.5 (3–540)	100.86 ± 61.99	92 (3–780)	0.184**
Femoral puncture- to microcatheter time (min.)	18.67 ± 11.07	15 (4–50)	20.39 ± 14.4	17 (4–126)	20.19 ± 14.04	17 (4–126)	0.589**
Femoral puncture- to recanalization time (min.)	47.03 ± 30.51	40 (6–160)	46.51 ± 30.18	39.5 (10–232)	46.57 ± 30.16	40 (6–232)	0.856**
Discharge mRS	4.1 ± 2	5 (0–6)	2.98 ± 2.14	3 (0–6)	3.11 ± 2.15	3 (0–6)	0.001**
3-month mRS	3.93 ± 2.41	5 (0–6)	2.56 ± 2.36	2 (0–6)	2.73 ± 2.41	2 (0–6)	<0.001**

*Independent Two Sample t test; **Mann Whitney U testi; ICAD, intracranial atherosclerotic disease; NIHSS, National Institutes of Health Stroke Scale Score; mRS, modified Rankin Score. Bold values indicate statistically significant differences (*p* < 0.05).

**Table 2:** Baseline characteristics of the study populationa characteristics of the intracranial atherosclerotic disease and cardioembolic stroke groups.

Parameters	Characteristic	ICAD group	Cardioembolic Group	All patients	*p* value
Sex	Female	16 (38.1)	172 (56)	188 (53.9)	0.043*
	Male	26 (61.9)	135 (44)	161 (46.1)	
Comorbidities	Hypertension	26 (61.9)	174 (56.7)	200 (57.3)	0.634*
	Diabetes mellitus	13 (31)	71 (23.1)	84 (24.1)	0.357*
	Coronary artery disease	5 (11.9)	113 (36.8)	118 (33.8)	0.002*
	Atrial fibrillation	2 (5)	189 (62.2)	191 (55.5)	<0.001*
	Previous stroke	10 (23.8)	55 (17.9)	65 (18.6)	0.478*
	Active cancer	0 (0)	7 (2.3)	7 (2)	1.000**
Risk factors	Obesity	10 (23.8)	49 (16)	59 (16.9)	0.292*
	Current smoking	15 (35.7)	48 (15.6)	63 (18.1)	0.003*
	Chronic alcoholism	1 (2.4)	5 (1.6)	6 (1.7)	0.539**
	Pregnancy	0 (0)	3 (1.8)	3 (1.6)	1.000**
Prior drugs	Antiaggregant	11 (26.2)	98 (31.9)	109 (31.2)	0.566*
	DOAC	0 (0)	44 (14.4)	44 (14.4)	0.017*
IV rtPA + EVT	IV rtPA administered	16 (38.1)	133 (43.3)	149 (42.7)	0.634*
	IV rtPA contraindicated	15 (37.5)	116 (38)	131 (38)	1.000*
	IV rtPA full dose	3 (18.8)	38 (28.6)	41 (27.5)	0.558**
	IV rtPA not full dose	13 (81.3)	95 (71.4)	108 (72.5)	
IV rtPA Reasons for Contraindication	Patient arrives 4.5 h later	7 (46.7)^a^	16 (13.8)^b^	23 (17.6)	<0.001***
	Symptom time is not clear	4 (26.7)^a^	7 (6)^b^	11 (8.4)	
	Waking stroke	1 (6.7)	7 (6)	8 (6.1)	
	Major surgical procedure performed	0 (0)	8 (6.9)	8 (6.1)	
	History of stroke within 3 months	2 (13.3)^a^	3 (2.6)^b^	5 (3.8)	
	Warfarin use	0 (0)^a^	27 (23.3)^b^	27 (20.6)	
	Anticoagulant use in the last 24 h	0 (0)^a^	30 (25.9)^b^	30 (22.9)	
	Other	1 (6.7)	18 (15.5)	19 (14.5)	
Radiological imaging	Hyperdense artery sign	19 (46.3)	207 (68.8)	226 (66.1)	0.008*
	Anterior circulation	24 (57.1)	291 (94.8)	315 (90.3)	<0.001**
	Posterior circulation	18 (42.9)	16 (5.2)	34 (9.7)	
Collateral score (TAN)	None	3 (12.5)	15 (5.8)	18 (6.3)	0.296***
	<%50	7 (29.2)	119 (45.8)	126 (44.4)	
	>%50	13 (54.2)	111 (42.7)	124 (43.7)	
	Same or More than Opposite	1 (4.2)	15 (5.8)	16 (5.6)	
Collateral score (modified TAN)	Good	15 (62.5)	129 (49.6)	144 (50.7)	0.320*
	Poor	9 (37.5)	131 (50.4)	140 (49.3)	

*Yates correction; **Fisher’s Exact test; ***Pearson Chi- Square test; ^a-b^There is no difference between groups with the same letter. ICAD, intracranial atherosclerotic disease; IV rtPA intravenous recombinant tissue plasminogen activator; EVT, endovascular treatmenty; DOAC, direct oral anticoagulant. Bold values indicate statistically significant differences (*p* < 0.05).

### Endovascular treatment

In the ICAD group, recanalization resulted in good outcome (mTICI 2c-3) in 22 (52.4%) patients, while recanalization resulted in poor outcome (mTICI 0–2b) in 20 (47.6%) patients. In the cardioembolic group, recanalization resulted in good outcome (mTICI 2c-3) in 188 (61.2%) patients, while recanalization resulted in poor outcome (mTICI 0–2b) in 119 (38.8%) patients. There was no statistically significant difference between the two groups (*p* = 0.352).

Propensity score matching was conducted with a tolerance level of 0.200. As a result, 40 matched cases were successfully obtained from the cardioembolism group, corresponding to the 42 individuals in the ICAD group. Across a total of 12.587 matching attempts, a 24.4% rejection rate was observed. According to the matching results, an average of 26.95 suitable matches were available per case. These findings suggest that the applied tolerance level effectively achieved a sufficient number and quality of matches.

After PSM, the proportion of good outcomes (mTICI 2c–3) in the ICAD group remained unchanged at 52.4%. In contrast, among the 40 randomly selected patients in the cardioembolic group after PSM, only 16 (40%) achieved good outcomes (mTICI 2c–3), whereas the remaining 24 patients (60%) had poor outcomes (mTICI 0–2b). Thus, following PSM, the proportion of good recanalization outcomes in the cardioembolic group decreased from 61.2% (pre-PSM) to 40%. The odds of achieving good clinical outcome (mRS 0–2) were found to be OR = 3.656 (95% CI, 1.460–9.156) times higher in the successful reperfusion (mTICI) group than in the unsuccessful reperfusion (mTICI) group. Pearson chi-square test showed that this difference was significant (χ^2^(1) = 7.947, *p* = 0.005). Ordinal logistic regression showed that higher mRS scores at discharge or 3 months were significantly associated with lower mTICI scores (*p* = 0.001). Each unit increase in discharge mRS reduced the odds of a higher mTICI category by 31% (OR = 0.69, 95% CI: 0.58–0.84), while a one-point increase in 3-month mRS led to a 26% decrease (OR = 0.74, 95% CI: 0.63–0.88).

There was a statistically significant correlation between the groups in terms of intracranial angioplasty (*p* < 0.001). The rate of intracranial angioplasty in the ICAD group was 35.7%, while this rate was 2.3% in the cardioembolic stroke group. The probability of outcomes was significantly higher also after PSM (OR = 21.67, 95% CI: 2.70–173.93). There was a statistically significant correlation between the groups in terms of permanent intracranial stent (*p* < 0.001). In the ICAD group, the rate of those who underwent permanent intracranial stenting during the procedure was 26.2%, while this rate was 1.6% in the cardioembolic stroke group. The probability of outcomes was significantly higher also after PSM (OR = 13.84, 95% CI: CI: 1.69–113.09). The rate of reocclusion in the angiography unit after mechanical thrombectomy was statistically significant between the groups (*p* = 0.002). The rate of reocclusion in the angiography unit after mechanical thrombectomy was 14.3% in the ICAD group and 2.3% in the cardioembolic group. Even after propensity score matching, a statistically significant difference remained between the two groups, with an odds ratio of 1.167 (95% CI: 1.031–1.320).

### Procedure complications

Post-PSM crosstabulation showed that vasospasm after EVT occurred in 0 patients in the cardioembolism group (0/40) and in 1 patient in the ICAD group (1/42, 2.4%). No statistically significant association was found between the groups.

CT imaging within 24 h post-EVT revealed hemorrhage in 6 (14.3%) patients in the ICAD group and 75 (24.4%) patients in the cardioembolic stroke group. Clinical progression (symptomatic intracranial hemorrhage) caused by bleeding after EVT was seen in 3 (27.3%) patients in the ICAD group and in 20 (19.6%) patients in the cardioembolic stroke group. The results were not statistically significant. When other parameters were analyzed, no statistically significant relationship was found between the groups (*p* > 0.050; Table 3). Statistical significance after PSM was found to be similar to that before PSM. After PSM, analysis showed that within 24 h after EVT, hemorrhage was observed in 11 cardioembolic patients (27.5%). Statistical analysis with Chi-Square test indicated no significant association between stroke etiology and the presence of hemorrhage (*p* = 0.140). There was no significant association between stroke etiology and post-EVT symptomatic intracerebral hemorrhage (*p* = 0.892). In contrast to the findings before PSM, dissection during EVT was significantly more frequent in the ICAD group after PSM (OR: 1.105, 95% CI: 1.002–1.219).

**Table 3:** Procedure results of intracranial atheroscrotic disease and cardioembolic stroke groups.

Parameters		ICAD Group	Cardioembolic Group	All patients	*p*-value
First pass recanalization		15 (35.7)	133 (43.8)	148 (42.8)	0.412*
Second pass recanalization		5 (11.9)	47 (15.5)	52 (15)	0.708*
Cases with number of procedures ≥3		23 (54.8)	145 (47.7)	168 (48.6)	0.488*
Patients recanalized in the first 45 min		21 (50)	156 (51.3)	177 (51.2)	1.000*
Intra arterial rtPA was administered		3 (7.3)	53 (17.4)	56 (16.2)	0.157*
Intracranial angioplasty		15 (35.7)	7 (2.3)	22 (6.3)	<0.001**
Permanent intracranial stent		11 (26.2)	5 (1.6)	16 (4.6)	<0.001**
Tirofiban infusion after permanent stent		10 (23.8)	5 (1.6)	15 (4.3)	<0.001**
Hemorrhage on CT in the first 24 h after EVT		6 (14.3)	75 (24.4)	81 (23.2)	0.206*
Hemorrhagini-related clinical progression after EVT		3 (27.3)	20 (19.6)	23 (20.4)	0.693**
Post-procedure recanalization scale (mTICI)	mTICI 0	2 (4.8)	7 (2.3)	9 (2.6)	0.714***
	mTICI1	1 (2.4)	3 (1)	4 (1.2)	
	mTICI2a	6 (14.3)	38 (12.6)	44 (12.8)	
	mTICI2b	11 (26.2)	65 (21.5)	76 (22.1)	
	mTICI2c	10 (23.8)	100 (33.1)	110 (32)	
	mTICI3	12 (28.6)	89 (29.5)	101 (29.4)	
Distal embolism after EVT		19 (45.2)	160 (52.6)	179 (51.7)	0.463*
Vascular rupture during EVT		1 (2.4)	3 (1)	4 (1.2)	0.406**
Dissection during EVT		4 (9.5)	15 (4.9)	19 (5.5)	0.266**
Reocclusion after EVT		6 (14.3)	7 (2.3)	13 (3.8)	0.002**
Vasospasm after EVT		1 (2.4)	96 (31.6)	97 (28)	<0.001*
Decompression after EVT		2 (4.8)	22 (7.2)	24 (6.9)	0.752**
Hemorrhage on CT in the first 24 h after EVT		6 (14.3)	75 (24.4)	81 (23.2)	0.206*
Type of hemorrhage on CT after EVT	Type 1 petechial	0 (0)	20 (26.7)	20 (24.7)	0.793***
	Type 2 petechial	1 (16.7)	9 (12)	10 (12.3)	
	Type 1 hematoma	3 (50)	27 (36)	30 (37)	
	Type 2 hematoma	1 (16.7)	7 (9.3)	8 (9.9)	
	Subarachnoid Hemorrhage	1 (16.7)	11 (14.7)	12 (14.8)	
	Distant hematoma unrelated to infarct	0 (0)	1 (1.3)	1 (1.2)	
Good outcomes		14 (33.3)	169 (55)	183 (52.4)	0.013*
Poor outcomes		28 (66.7)	138 (45)	166 (47.6)	

*Yates correction; **Fisher’s Exact test; ***Pearson Chi- Square test. EVT, endovascular treatmenty. Bold values indicate statistically significant differences (*p* < 0.05).

### Clinical outcome

Patients with ICAD had significantly higher mean 3-month mRS scores (3.93 vs. 2.78) and discharge mRS scores (4.10 vs. 3.13) compared to those with cardioembolic group (*p* < 0.05, both 3-month and discharge mRSs). Effect sizes (Cohen’s d = 0.48–0.49) indicated a moderate difference between groups. The distributions of both 3-month mRS and discharge mRS scores were significantly different between the ICAD and cardioembolic groups (*p* < 0.05; Table 4).

**Table 4:** Logistic regression analysis performed on ICAD and cardioembolic stroke groups following propensity score matching.

Variable	ICAD (*n* = 42)	Cardioembolic (*n* = 40)	Effect estimate	95% CI	*p*-value
Intracranial angioplasty	35.7%	2.3%	OR = 21.67	2.70–173.93	**< 0.001**
Permanent intracranial stent	26.2%	1.6%	OR = 13.84	1.69–113.09	**< 0.001**
Reocclusion after EVT	14.3%	2.3%	OR = 1.17	1.03–1.32	**0.002**
Discharge mRS (mean)	4.10	3.13	Mean diff = 0.97 (Cohen’s d = 0.49)	—	**< 0.05**
3-month mRS (mean)	3.93	2.78	Mean diff = 1.15 (Cohen’s = 0.48)	—	**< 0.05**

ICAD, intracranial atherosclerotic diseases; EVT, endovascular treatment; mRS, modified Rankin score. Bold values indicate statistically significant differences (*p* < 0.05).

Logistic regression was conducted specifically on the poor-outcome (mTICI = 0–2b) group (*n* = 44). In this subgroup, the 3-month mRS score was a significant independent predictor of stroke etiology (ICAD vs. cardioembolic), with OR of 1.53 (95% CI: 1.08–2.17, *p* = 0.016). The model demonstrated an acceptable goodness-of-fit (Hosmer–Lemeshow test *p* = 0.943), with an overall classification accuracy of 68.2% and a Nagelkerke R^2^ of 0.192.

In summary, among the subgroup of patients with mTICI = 0–2b, a higher 3-month mRS score emerged as the most significant, consistent, and reliable predictor for distinguishing ICAD from cardioembolic stroke.

**Figure 1:**
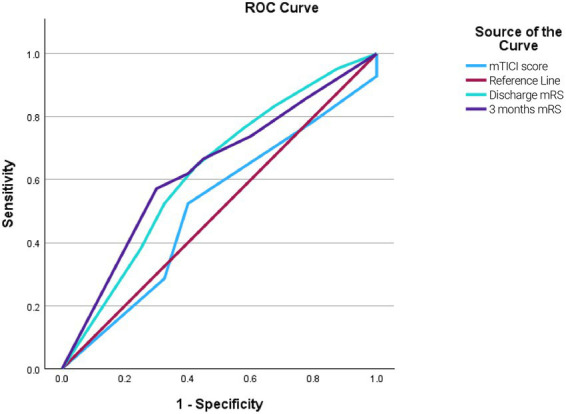
ROC curve analysis: The ROC curves illustrate the discriminatory performance of the mTICI score (blue), discharge mRS (cyan), and 3-month mRS (purple), with the red line indicating the reference (random chance) line.

The area under the ROC curve (AUC) was 0.631 for 3-month mRS, 0.510 for mTICI score, and 0.626 for discharge mRS, indicating low to moderate discriminatory ability for these variables (Figure 1).

**Figure 2:**
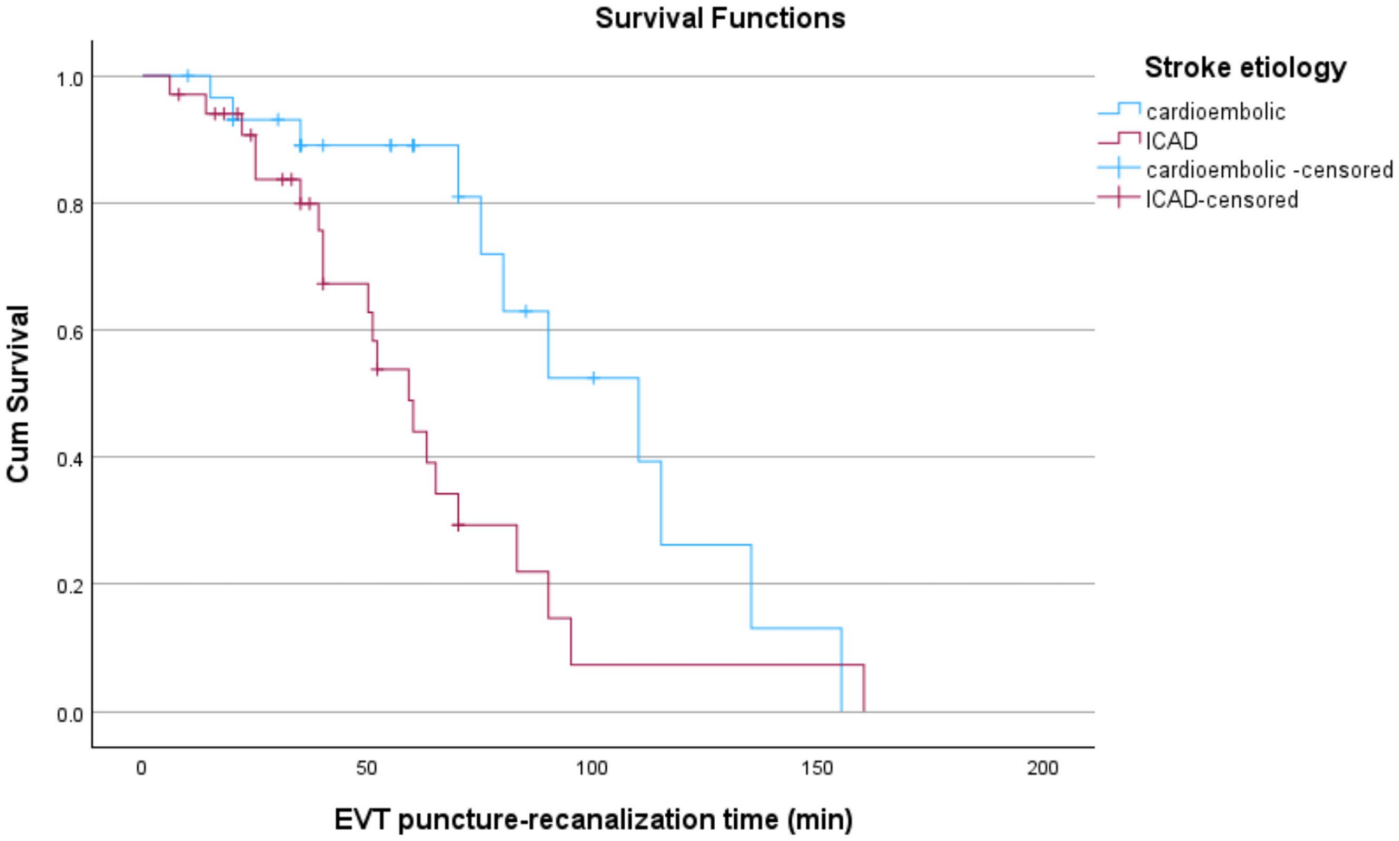
Kaplan–Meier curves comparing EVT puncture-to-recanalization times by stroke etiology. ICAD patients show significantly shorter times than those with cardioembolic strokes. Censored data points are marked with “+”.

Patients with cardioembolic stroke had a notably longer median survival time of 110 min (95% CI: 70.55–149.45) compared to 59 min (95% CI: 46.28–71.72) in patients with ICAD stroke. Censoring rates were higher in the cardioembolic group (63.3%) compared to the ICAD group (38.2%), indicating fewer recorded events in the cardioembolic group by the study endpoint. The survival distributions between stroke etiologies (ICAD vs. cardioembolism) significantly differed according to all three statistical tests: Log Rank (Mantel-Cox; χ^2^(1) = 6.387, *p* = 0.011), Breslow (Generalized Wilcoxon; χ^2^(1) = 6.173, *p* = 0.013), and Tarone-Ware (χ^2^(1) = 7.581, *p* = 0.006). These findings suggest a statistically significant survival advantage for patients with cardioembolic group compared to those with ICAD etiologies (Figure 2).

## Discussion

In this study, which analyzed the outcomes of ICAD-related stroke patients, a condition less common in the Turkish population and cardioembolic stroke patients, the proportion of patients in the ICAD group was lower than in the cardioembolic group. There was no statistically significant difference in age between the two groups in our study. When the literature is reviewed, it has been shown that Asian patients with ICAD are generally younger than those in Western countries (6). It has been reported that male predominance in ICAD compared to embolic etiologies, as well as young age, smoking, and alcohol consumption are higher in the Asian population (7). In our study, the proportion of males in the ICAD group was found to be higher in accordance with the literature.

Active smoking has been reported to be more frequent in acute stroke patients with ICAD compared to cardioembolic stroke patients (8). Similar results were found in our study.

In a study in which cardioembolic strokes were examined, it was shown that the patients were older, and the rate of women was higher compared to men. In addition, it was reported that comorbid conditions, including cardiovascular disease, hypercholesterolemia, atrial fibrillation, and smoking, were also higher in this patient group (9). In this study, it was reported that NIHSS mean scores at the time of presentation were higher in the cardioembolic stroke group (9).

In another study, it was shown that patients with ICAD-related large vessel occlusion had a lower baseline NIHSS score and a higher ASPECT score compared with embolism-induced stroke patients (7). In our study, no statistically significant difference was found between the two groups in terms of baseline NIHSS score and ASPECT score.

Lee et al. emphasized in a study that the rate of acute stroke patients with posterior circulation involvement was high in the ICAD group (6). Basilar artery occlusion accounts for approximately 10% of all ischemic strokes caused by intracranial proximal large vessel occlusion. These patients have high morbidity and mortality (10, 11).

In a cohort study, it was emphasized that 10% of ICAD were anterior circulatory system infarcts, and 40% were posterior circulatory system infarcts. Although these rates are high, unfortunately, the efficacy of mechanical thrombectomy procedures has mostly been evaluated in patients with occluded anterior circulatory system. Whether these procedures can also work in posterior circulatory occlusions has not been adequately evaluated (6).

In a multicentre study of patients with acute basilar artery occlusion, the outcomes of patients with embolic causes were compared with those of ICAD patients. It was emphasized that successful recanalisation rates were lower in ICAD patients, while morbidity and mortality rates were higher. No significant difference was found between the two groups in terms of symptomatic intracranial haemorrhage rates (12).

In our study, the rate of strokes with posterior system occlusion was 42% in the ICAD group, whereas this rate was 5% in the cardioembolic stroke group. Consistent with the literature, the presence of atrial fibrillation was found to be higher in the cardioembolic group in our study (6).

Although reperfusion is initially established after EVT in patients with ICAD, reocclusions may often develop due to platelet aggregation (6). In our study, the rate of reocclusion was higher in the ICAD group (14%) compared to the cardioembolic stroke group (2%). Furthermore, after propensity score matching, arterial dissection during EVT was significantly more frequent in the ICAD group.

Extensive studies in the literature have shown that acute intracranial angioplasty and stenting may be an appropriate salvage treatment for underlying ICAD in acute stroke patients with large vessel occlusion in whom EVT failed (1, 8, 13). In our study, the rate of permanent intracranial stenting as salvage treatment after EVT in the ICAD group was 26%, and the rate of intracranial angioplasty was 35%. Our results were statistically significant.

In acute stroke patients with ICAD, rescue therapy requires rapid antiplatelet administration after stenting. Although intra-arterial tirofiban infusion seems to be appropriate in the treatment of ICAD-related occlusions, safe data are still limited in these patient groups receiving IV rtPA before EVT (6, 14, 15). Intracranial hemorrhage rate in patients with ICAD treated with glycoprotein 2a/3b inhibitors has been shown to be around 4% (8). In our study, the rate of ICAD patients who received intraarterial tirofiban infusion after permanent stenting was 24%. Recent studies in the literature have shown that salvage therapy does not increase the risk of intracranial hemorrhage and death in stroke patients with ICAD who underwent EVT but improves the recanalization rate and clinical outcome (13).

In cardioembolic stroke patients, it has been reported that the longer the time from femoral puncture to the end of the procedure, the lower the rate of good outcome, but this was not observed in patients with large vessel atherosclerosis. There are studies emphasizing that good collateral circulation contributes to good clinical outcomes in acute stroke patients undergoing EVT (16–19). In our study, good collateral circulation (mTAN) was 62% in the ICAD group and 49% in the cardioembolic stroke group. The presence of good collateral circulation did not have a significant impact on patient outcomes.

In our study, there was no statistically significant difference between the two groups in femoral puncture-to-recanalization time. It has been reported that *in situ* thromboses cause longer EVT durations and worse outcomes in ICAD-related acute stroke patients (1).

In a multicenter, prospective study evaluating outcomes of patients with intracranial atherosclerotic disease (ICAD) and cardioembolic stroke, it was reported that in the ICAD group recanalization times were longer, and recanalization success was lower (20).

In our study, the rate of patients with ICAD-related stroke who ended with a poor outcome in the early 3 months after EVT was found to be 66%. This rate was 45% in patients with cardioembolic stroke etiology.

In a recent study evaluating the outcome of thromboembolic stroke and ICAD-related acute stroke patients, it was shown that there was no significant difference between EVT procedure duration, successful recanalization, 90-day mRS results, and mortality rates (4).

Another study evaluating the outcomes of patients with large artery atherosclerosis and cardioembolic stroke showed no difference between the groups in terms of symptomatic intracranial hemorrhage and mortality. The recanalization rate and 90th-day functional independence (mRS0-2) rates of the two groups were shown to be similar (7, 8, 18). Another study showed that 3-month survival was worse in cardioembolic stroke patients (9). In our study, 3-month functional independence (mRS0-2) rates were 33% in the ICAD group and 55% in the cardioembolic stroke group. In our study, the rate of patients with bleeding on CT in the first 24 h after EVT was 14% in the ICAD group and 24% in the cardioembolic stroke group. When the clinical progression caused by bleeding was analyzed, the rate of symptomatic intracranial hemorrhage was 27% in the ICAD group and 19% in the cardioembolic stroke group. Although the results were not statistically significant, the rate of symptomatic intracranial hemorrhage was higher in the ICAD group than in the cardioembolic group.

In a study comparing the outcomes of patients with underlying ICAD and patients with other stroke subtypes, it was shown that acute stroke patients presenting in the hyperacute period in the ICAD group had a lower stroke severity (NIHSS value) at presentation, a higher rate of successful revascularization after multimodal EVT and a lower clot burden (21).

In a study published in 2021, Yang et al. reported no significant difference in the rate of post-procedural intracranial hemorrhage between ICAD-related and cardioembolic stroke patients. Interestingly, they found that complete recanalization (mTICI 3) rates were higher in patients with ICAD-related stroke (7). In contrast, the STRATIS study, which included 978 patients, found that ICAD-related strokes accounted for 9.3% (*n* = 91) and cardioembolic strokes for 39% (*n* = 379) of the cohort. Among the 665 patients with complete angiographic imaging data, successful reperfusion rates were significantly lower in ICAD-related stroke compared to cardioembolic stroke, highlighting some variability across studies (22).

In our study, after propensity score matching, the rate of successful recanalization (defined as mTICI 2c–3) was 52% in the ICAD group and 40% in the cardioembolic group. However, this difference was not statistically significant. Notably, while the ICAD group size remained unchanged (*n* = 42) following matching, only 40 cardioembolic patients could be matched based on similar baseline characteristics. This matching process may have led to the selection of a cardioembolic subgroup with less favorable clinical or radiological features, such as longer onset-to-treatment time, higher clot burden, or poorer collateral circulation, compared to the unmatched cardioembolic population. This may partially explain the notable decrease in recanalization rates in the cardioembolic group (from 61.2 to 40%).

### Study limitations

One of the main limitations of this study is its single-center and retrospective design. Additionally, outcome assessments were not blinded, as assessors had access to clinical information, which may have introduced observer bias. Another limitation is the significant imbalance in the number of patients between the two groups. This discrepancy is primarily due to the lower prevalence of ICAD-related stroke in the Turkish population compared to East Asian populations.

## Conclusion

In acute stroke patients, the underlying ICAD cannot be easily predicted before EVT. This makes it difficult to plan treatment and pharmacologically prepare the patient before intervention. Early prediction of ICAD may help us to change the treatment approach before starting the intervention. Parameters such as stroke severity, collateral status, the appearance of hyperdense arteries, localization of occlusion, etc., should be taken into consideration. Obviously, anterior circulation occlusion is mostly found in cardioembolic strokes, whereas posterior circulation occlusions are more common in the ICAD group. This difference was similar in both the pre-PSM measurements in 349 patients and the post-PSM measurements in 82 patients. Hyperdense artery appearance is more common in cardioembolic strokes. Collateral circulation is well developed in ICADs compared to cardioembolic strokes. Thrombectomy may be more difficult to perform in the ICAD group. Reocclusion is more likely to occur in the ICAD group. The increased number of intracranial procedures, intracranial stenting, balloon angioplasty, and administration of tirofiban infusion indicate that symptomatic bleeding is more common in ICAD patients. Increasing number and duration of procedures leads to the risk of poor outcomes in the ICAD group.

## Data Availability

The original contributions presented in the study are included in the article/supplementary material, further inquiries can be directed to the corresponding author.
